# Chemical Constituents, Anti-Tumor Mechanisms, and Clinical Application: A Comprehensive Review on *Scutellaria barbata*

**DOI:** 10.3390/molecules29174134

**Published:** 2024-08-31

**Authors:** Jiagui Sun, Yuqi Cao, Qiqi Liu, Zhengshu Zhou, Yanan Xu, Chenggang Liu

**Affiliations:** School Basic Medical Sciences, Heilongjiang University of Chinese Medicine, 24 Heping Road, Harbin 150040, China; 13209846730@139.com (J.S.); qq1656445881@163.com (Y.C.); liuqiqi0862@163.com (Q.L.); zhzsyydsh@163.com (Z.Z.); 15838399049@163.com (Y.X.)

**Keywords:** *Scutellaria barbata* D. Don, chemical constituents, anti-tumor mechanisms, clinical application

## Abstract

With the increasing global incidence and mortality rates of cancer, the development of novel anti-tumor drugs has become particularly urgent. *Scutellaria barbata* D. Don, a perennial herb belonging to the genus Scutellaria in the family Lamiaceae, has aroused extensive attention for its medicinal value in recent years. This article presents an exhaustive review of the flavonoid, diterpene, and other chemical constituents harbored within *Scutellaria barbata*, delving into the intricate mechanisms by which these compounds orchestrate their anti-tumor effects via diverse biological pathways. Remarkably, these compounds distinguish themselves through their capability to regulate cellular signaling, inhibit cancer cell proliferation, trigger apoptosis, disrupt angiogenesis, and bolster immune responses. These anti-tumor effects are achieved through strategic modulation of pivotal signaling cascades, particularly the PI3K/Akt/mTOR, MAPK, and NFκB pathways. In addition, this article also summarizes the clinical applications of *Scutellaria barbata* in tumor treatment, especially its potential in alleviating the side effects of radiotherapy and chemotherapy and improving patients’ quality of life. In conclusion, this review comprehensively summarizes and analyzes the chemical constituents, anti-tumor mechanisms, and clinical applications of *Scutellaria barbata*, with the aim of systematically reviewing the existing research results and exploring potential future research directions.

## 1. Introduction

Cancer poses a significant threat to human health due to its difficulty in early diagnosis, high recurrence rates, and poor treatment outcomes. According to data from the World Health Organization (WHO), the incidence and mortality rates of cancer are increasing globally, with millions of people dying from various types of cancer each year. To date, the available cancer treatments include surgery, radiotherapy, chemotherapy, and targeted therapy. Although surgical resection is considered as the only effective treatment, many advanced cancer patients still require chemotherapy to reduce tumor growth or inhibit metastasis. However, due to the limited tumor-specific cytotoxic effects of existing drugs and the presence of resistance mechanisms in tumor cells, few advanced cancer patients benefit from chemotherapy, resulting in low long-term survival rates [1]. Traditional Chinese herbal medicine has a history of thousands of years of clinical application in China, accumulating rich experience and knowledge. Additionally, Chinese medicine contains numerous unknown components with potential pharmacological activity and therapeutic potential. Using modern technology to discover new drug molecules from Chinese medicine and develop them into new medications has shown great potential in the field of cancer treatment [2].

*Scutellaria barbata* D. Don (also known as Ban Zhi Lian) is a perennial herb of the Lamiaceae family, widely distributed in East Asia, including China, Japan, and Korea, and has been widely used in traditional medical systems in these countries. According to the literature, *Scutellaria barbata* has been used medicinally for hundreds of years, firstly documented in “Authentic Surgery” by the Ming Dynasty physician Chen Shigong, where it was noted for its efficacy in treating snake bites [3]. In the traditional Chinese medicine system, *Scutellaria barbata* is known for its effects of clearing heat and detoxifying, promoting diuresis to reduce swelling, and activating the blood circulation to resolve stasis. Additionally, it is often used alone or in combination with other herbs, such as *Hedyotis diffusa* (Bai Hua She She Cao), to treat tumors [4]. In the modern Chinese medicine system, *Scutellaria barbata* is included in the 2020 edition of the Chinese Pharmacopoeia, indicated for conditions like sores and abscesses, sore throat, traumatic injuries, edema, jaundice, and snake bites. Currently, there are proprietary Chinese medicines like *Scutellaria barbata* capsules and tablets for treating sore throat caused by acute pharyngitis. Moreover, many compound preparations contain *Scutellaria barbata*, such as Kang Gu Sui Yan Pian, Re Yan Ning He Ji, and Compound Banmao Capsule. With the advancement of modern medical research, the efficacy of *Scutellaria barbata* in cancer treatment has gained widespread attention from the scientific community.

Modern research on Chinese medicine has identified nearly 300 compounds from *Scutellaria barbata* through various extractions and separation methods, with the main ones being flavonoids and diterpenoids, along with polysaccharides, volatile compounds, trace elements, and steroids [5]. Flavonoids, the primary active components of *Scutellaria barbata*, include quercetin, luteolin, scutellarein, scutellarin, wogonin, and baicalein [6]. The terpenoids are mainly neo-clerodane diterpenoids, which have potential applications in immunomodulation and anti-tumor activities, and also show some antibacterial and antiviral activity [5].

*Scutellaria barbata* has demonstrated good hepatoprotective effects in clinical settings and was initially used as an adjuvant treatment for liver cancer. With deeper research into its components, *Scutellaria barbata*’s active ingredients have shown efficacy against malignant tumors such as breast cancer, ovarian cancer, prostate cancer, and leukemia [7,8,9,10,11,12,13,14]. Modern pharmacological studies have shown that *Scutellaria barbata* extracts, rich in flavonoids, can exert anti-tumor effects by inhibiting tumor cell proliferation, inducing apoptosis, inhibiting cell migration and invasion, suppressing angiogenesis, and modulating immunity [15]. Combining traditional Chinese medicine with chemotherapy is a distinctive feature of cancer treatment in China. *Scutellaria barbata* can enhance the efficacy of anti-tumor drugs and exhibit significant potential in overcoming drug resistance by inhibiting drug efflux pumps and regulating apoptosis [16,17]. Additionally, its anti-inflammatory properties can improve the tumor microenvironment and inhibit tumor progression. *Scutellaria barbata* exerts its anti-tumor effects by regulating multiple signaling pathways. It can inhibit the PI3K/Akt/mTOR pathway, reducing tumor cell growth and survival. The active components of *Scutellaria barbata* can inhibit the MAPK signaling pathway, including ERK, JNK, and p38, suppressing tumor cell proliferation and survival. Furthermore, it can inhibit the NFκB pathway, reducing the production of pro-inflammatory cytokines, alleviating inflammation, and thereby inhibiting tumor cell growth [4,18].

Currently, research on *Scutellaria barbata* has made some progress, though most studies remain at the laboratory stage, with relatively few clinical application studies. This review summarizes the clinical application of *Scutellaria barbata* in cancer treatment, providing an intuitive assessment of its therapeutic effects. Presently, the clinical application of *Scutellaria barbata* is mainly focused on adjuvant cancer therapy, particularly in alleviating side effects of radiotherapy and chemotherapy and improving patients’ quality of life.

This review aims to comprehensively summarize and analyze the chemical constituents, anti-tumor mechanisms, and clinical applications of *Scutellaria barbata*, systematically organizing the existing research outcomes and exploring future research directions. By summarizing the progress in the chemical composition and anti-tumor mechanisms of *Scutellaria barbata*, this review not only helps evaluate its potential application value in various cancers but also provides scientific evidence for its application in modern medicine, serving as a reference for future research and application. Despite the promising anti-tumor potential demonstrated by *Scutellaria barbata* in laboratory studies, further research is needed to verify its safety and efficacy in clinical applications.

## 2. Chemical Constituents

Numerous studies have reported on the chemical constituents of *Scutellaria barbata*, primarily including flavonoids, terpenoids, polysaccharides, volatile compounds, and trace elements. As research into *Scutellaria barbata* deepens, an increasing number of components are being isolated and identified. This section reviewed the research progress on the chemical constituents of *Scutellaria barbata* over the past decade, providing a reference for further research and the development of new therapeutic agents derived from *Scutellaria barbata*.

### 2.1. Flavanoids

**Figure 1:**
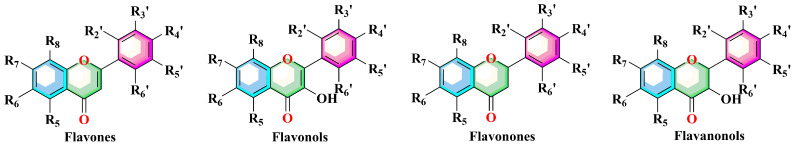
Core structure of flavones, flavonols, flavonones, and flavanonols in *Scutellaria barbata*.

**Figure 2:**
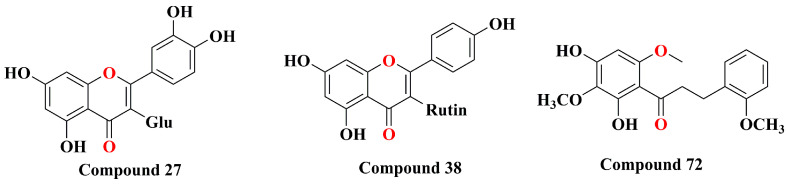
Structure of compound **27**, **38**, and **72**.

Flavonoids are viewed as a significant chemical component of *Scutellaria barbata*, and modern pharmacological studies have demonstrated their relevant anti-tumor activity. Numerous extraction methods for flavonoids have been reported, including solvent extraction, microwave-assisted extraction, supercritical fluid extraction, and acid–alcohol precipitation [19]. As shown in Figure 1, flavonoids can be classified into flavones, flavonols, flavanones, flavanonols, and chalcones, based on their core structures. According to Table 1 and Figure 2, over seventy flavonoids have been isolated and identified from *Scutellaria barbata*, including forty-seven flavones, three flavonols, fourteen flavanones, seven flavanonols, and one chalcone. Many active flavonoid components exist in glycoside form. Experiments have shown that the main flavonoid components of *Scutellaria barbata* include scutellarin, scutellarein, quercetin, luteolin, apigenin, and wogonin. Additionally, the extraction and exploration of flavonoid components are ongoing. Zhou et al. reported full-scan (FS)–parent ions list (PIL)–higher energy collision-induced dissociation (HCD)–MS/MS (FS-PIL-HCD-MS/MS) was used to discover potential flavonoid components. Further separation and identification are expected to reveal new flavonoid structures [20].

### 2.2. Terpenoids

Terpenoids are the most abundant chemical constituents in *Scutellaria barbata*, exhibiting significant pharmacological activity. The primary components are diterpenoids, with over 160 identified compounds. Additionally, triterpenoids such as ursolic acid and oleanolic acid have also been isolated and identified.

#### 2.2.1. Diterpenoids

**Figure 3:**
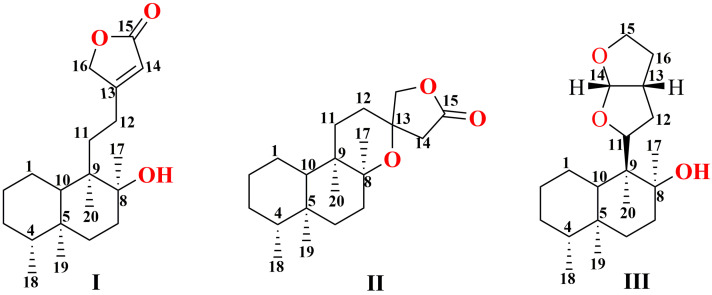
Three main core structure types of diterpenoids in *Scutellaria barbata*.

The main diterpenoids in *Scutellaria barbata* are primarily diterpenes and diterpene alkaloids, with their structures mainly classified as neo-clerodane diterpenoid. As Figure 3 shows, based on the variations in the side chain from C11 to C16, these diterpenoids can be categorized into three main classes. Class I features an unsaturated lactone ring formed between C13 and C16. Class II consists of compounds where C11–C16 and C8 form a spiro ring, usually existing in lactone form. Class III compounds feature a hexahydrofurano-furan ring type bicyclic structure formed between C11 and C16.

**Figure 4:**
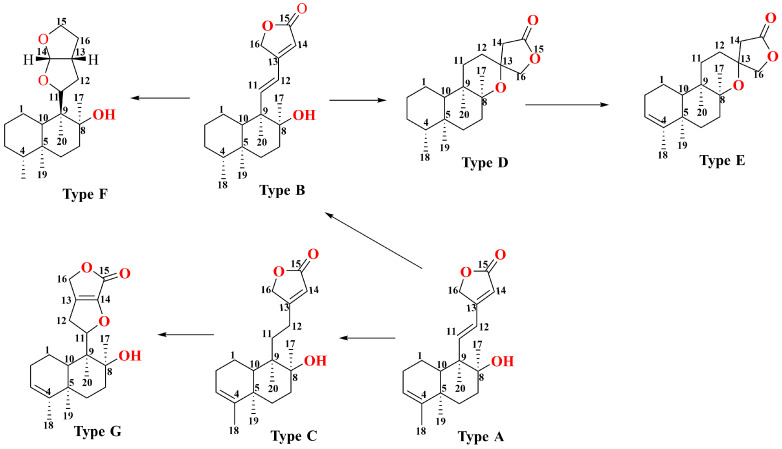
Side chain changes of the core structure of diterpenoids in *Scutellaria barbata*.

**Figure 5:**
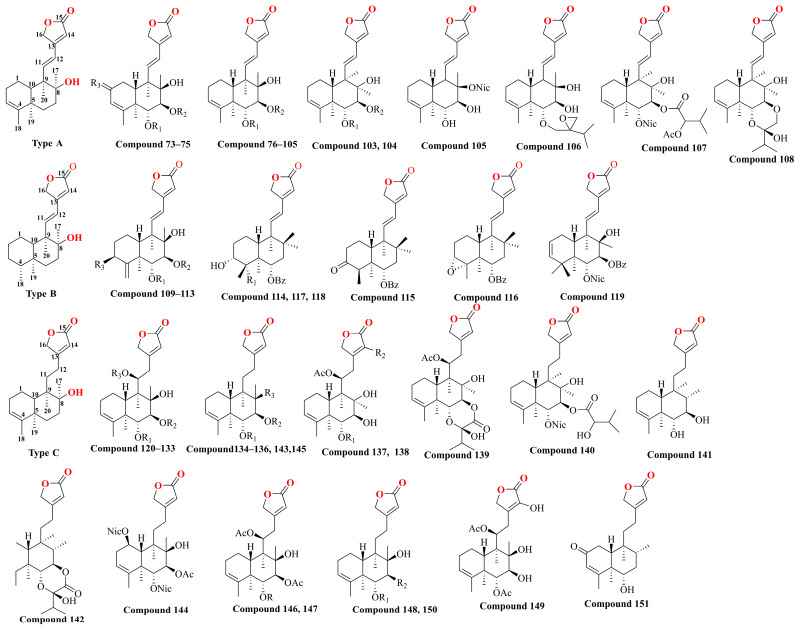

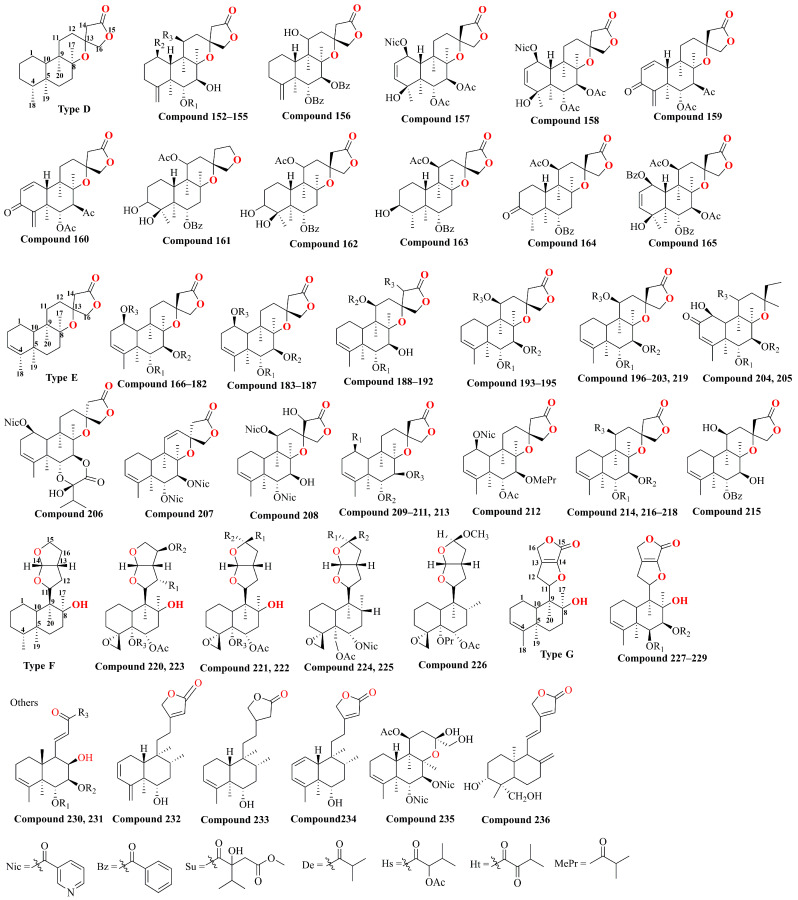
The chemical structure of diterpenoids isolated from *Scutellaria barbata*.

With further variations in the chemical structure of the side chains, these classes can be refined into seven structural types, as shown in Figure 4. The diversity in structural types and different substituent groups contributes to the large number of diterpenoid compounds. This section summarizes the diterpenoid compounds based on their structural types (Table 2 and Figure 5). Type A compounds are characterized by the cyclization of C13 to C16, forming an α-β unsaturated five-membered lactone. This type features a conjugated double bond between C11–C12 and C13–C14, and a double bond between C3–C4. Currently, 36 compounds of this type have been identified. When the C3–C4 double bond in Type A compounds was substituted with a single bond, the resulting compounds were classified as Type B. So far, 11 such Type B compounds have been systematically cataloged and definitively characterized. If the C11–C12 bond in Type A is a single bond, it is classified as a Type C compound, with 32 such compounds identified till now. Together, Type A, B, and C compounds constitute Class I, totaling 79 compounds. Type D compounds are characterized by a spirocyclic structure where C13 is linked to C8, forming a five-membered lactone structure with C13 as the spiro atom, and a double bond between C3–C4. To date, 14 compounds of this type have been identified. Type E compounds differ from Type D compounds in that the C3–C4 bond is a single bond. There are 54 such compounds. Combined with Type D, they form Class II, totaling 68 compounds. Type F compounds feature a bicyclic structure formed by a tetrahydrofuran and a furan ring, cyclizing from C11 to C16. Seven compounds of this type have been identified. Type G compounds are characterized by a spirocyclic lactone formed between C11–C16 and C8. This type is relatively rare, with only three compounds identified. Types F and G together constitute Class III, with a total of 10 compounds. In addition, there are 10 other types of compounds identified.

#### 2.2.2. Triterpenoids

Reports on triterpenoids in *Scutellaria barbata* are relatively limited. In 1988, Kuo et al. firstly isolated scutellarie acid from *Scutellaria barbata*. Subsequently, other triterpenoids such as stigmasterol, eleutheroside A, β-sitosterol, oleanolic acid, and ursolic acid have been identified [83,84,85]. 

### 2.3. Polysaccharides

In addition to flavonoids and terpenoids, *Scutellaria barbata* contains abundant polysaccharides distributed across different medicinal parts of the plant (whole plant, roots, stems, leaves, and flowers), with the highest concentration found in the roots. Xu et al. reported that the polysaccharide (PSB) has an average molecular weight of 13,000 Da and consists of six monosaccharides: rhamnose, arabinose, xylose, mannose, galactose, and glucose [86]. Meng et al. isolated and purified the SPS4 component, which comprises rhamnose, fucose, arabinose, xylose, mannose, glucose, and galactose, with a molar ratio of 0.22:0.26:1.0:0.09:0.51:1.82:2.09 [87]. Kong et al. extracted and identified acidic polysaccharide SPS II, and gas chromatography analysis of its hydrolysates revealed the presence of arabinose, mannose, fructose, and galactose [88]. Wu et al. obtained a homogeneous polysaccharide component, B3-PS2, with a molecular weight of 1100 kDa, primarily composed of glucose, galactose, and arabinose in a molar ratio of 2.7:2.7:1.0, along with small amounts of mannose, rhamnose, fucose, and xylose [89]. Wang et al. indicated that the monosaccharide composition of *Scutellaria barbata* polysaccharides includes rhamnose, fucose, arabinose, xylose, mannose, glucose, and galactose, with a molar ratio of 1.00:0.13:0.80:0.10:0.34:0.56:2.04 [90]. Sun et al. identified a polysaccharide, SPS2p, with monosaccharide composition of galactose, glucose, mannose, and arabinose, in a ratio of 1.59:2.59:1.00:1.31 [91]. Lin et al. discovered that the polysaccharide SBPW3 consists of glucose, arabinose, rhamnose, mannose, xylose, and galactose in a molar ratio of 20.59:25.68:2.51:12.56:10.94:27.72 [92]. Su et al. isolated two water-soluble polysaccharides, SBP-1A and SBP-2A, with monosaccharide compositions of galactosamine hydrochloride, rhamnose, arabinose, glucosamine hydrochloride, galactose, glucose, xylose, and mannose. The molar ratios of these components in SBP-1A were 0.6:0.3:0.6:30.6:2.3:38.4:16.1:8:1.4 and in SBP-2A were 0.6:0.5:0.8:36.3:4.4:42.7:9.2:2.6:0.7 [7]. Wu et al. isolated polysaccharides with an average molecular weight of 1.25 × 10^4^ Da, primarily composed of arabinose, galacturonic acid, galactose, glucose, and glucuronic acid in a molar ratio of 1.00:2.09:4.52:4.73:4.90 [93].

### 2.4. Volatile Compounds

*Scutellaria barbata* contains a rich variety of volatile oils with notable antimicrobial activity. Recent studies, primarily using GC-MS, have identified over 70 volatile components. Yu et al. isolated 41 volatile oil components, with the main compounds being hexahydrofarnesylacetone (11.0%), 3,7,11,15-tetramethyl-2-hexadecen-1-ol (7.8%), menthol (7.7%), and 1-octen-3-ol (7.1%) [94]. Zhang et al. identified 35 volatile oil components, with high relative contents of furfural (20.53%), thymol (24.10%), and palmitic acid (16.56%) [95]. Wang et al. identified 54 volatile oil components, with 11 compounds having contents above 1%, with the most abundant being palmitic acid (34.07%), linoleic acid (12.21%), and phytol (5.90%) [96]. Yang et al. identified 43 volatile oil components, with 10 compounds having contents above 1%, with the most abundant being n-palmitic acid (21.03%), (*Z*,*Z*)-9,12-linoleic acid (17.07%), trans-13-octadecenoic acid (14.10%), and stearic acid (2.52%) [97]. Differences in the volatile components can be attributed to factors such as the origin of *Scutellaria barbata*, harvest time, storage conditions, sample preparation, and analytical methods [97].

### 2.5. Trace Elements

*Scutellaria barbata* contains various trace elements such as Fe, Zn, Mn, Se, Ca, Mg, K, and Cu, with higher concentrations than many other traditional Chinese medicines [98,99]. Studies have shown significant positive correlations between the total flavonoid content and Zn content, and a very significant positive correlation with Mn content; polysaccharides also showed significant positive correlations with Zn and Mn contents [98]. These trace elements work synergistically in *Scutellaria barbata*, providing significant positive effects on human physiological functions.

### 2.6. Steroids

Steroids are a class of naturally occurring organic compounds widely distributed in nature. Using supercritical CO_2_ fluid extraction, steroidal components can be selectively extracted from *Scutellaria barbata*, with steroidal compounds accounting for 60% of the total components. The identified steroidal compounds include β-sitosterol, stigmasterol, campesterol, stigmast-4-en-3-one [100], Cholest-5-ene-3-thiol (3β)-Stigmastan-3,5,22-trien [97], and Daucosterol [101].

### 2.7. Phenylpropanoid and Lignan

Relatively fewer phenylpropanoids and lignans have been isolated from *Scutellaria barbata*. Currently, *E*−1-(4-Hydroxyphenyl)-but-1-en-3-one [102], 4-(3,4-dihydroxy-phenyl)-but-3-en-2-one [37], Chlorogenic acid [36], [(*E*)-3-(4-hydroxyphenyl)-acrylic acid ethyl] ester [31], pinoresinol, medioresinol [103], and d-syringaresinol [83] have been isolated.

### 2.8. Phenolic Acids

Various phenolic acids have been isolated from *Scutellaria barbata*, including benzoic acid, cinnamic acid [104], vanillic acid, isovanillic acid [105], 2,4-dihydroxybenzoic acid [35], and (*S*)-2-(4-hydroxyphenyl)-6-methyl-2,3-dihydro-4*H*-pyran-4-one [31].

### 2.9. Others

In addition to the aforementioned types of compounds, there are a few other types of compounds reported in *Scutellaria barbata*, such as coumarins and amides [106,107]. Due to their limited quantity, the pharmacological effects of these compounds in *Scutellaria barbata* are relatively less studied.

## 3. Anti-Tumor Mechanism

In traditional Chinese medicine theory, *Scutellaria barbata* is renowned for its efficacy in clearing heat, detoxification, diuresis, resolving swelling, promoting blood circulation, and removing blood stasis. Its therapeutic effects on liver diseases have also been widely reported. In recent years, *Scutellaria barbata* has attracted significant attention in oncology. With the advancement of modern medical technology, the mechanisms of its anti-tumor, anti-inflammatory, and antiviral effects have been gradually elucidated [6,48,59].

Research has shown that *Scutellaria barbata* and its extracts exert inhibitory effects on various cancer cells, including liver cancer [7,8,9,10,11,108], breast cancer [12,13,14], ovarian cancer [109], and prostate cancer [110]. The active components in *Scutellaria barbata* achieve anti-tumor effects through pathways such as the inhibition of cancer cell proliferation [111], induction of cancer cell apoptosis [9], suppression of tumor cell migration and invasion [12], and inhibition of tumor angiogenesis [8]. Among the identified chemical constituents of *Scutellaria barbata* extracts, flavonoids such as quercetin, luteolin, scutellarin, and wogonin have been extensively studied for their anti-tumor properties [8,112]. In clinical practice, in addition to being used independently for adjunctive cancer therapy, *Scutellaria barbata* is often combined with *Hedyotis diffusa* due to the latter’s effects in clearing heat, detoxification, promoting diuresis, resolving swelling, and dispersing masses. This combination exhibits synergistic effects and is widely applied in the adjuvant treatment of cancer.

### 3.1. Inhibition of Tumor Cell Proliferation

**Figure 6:**
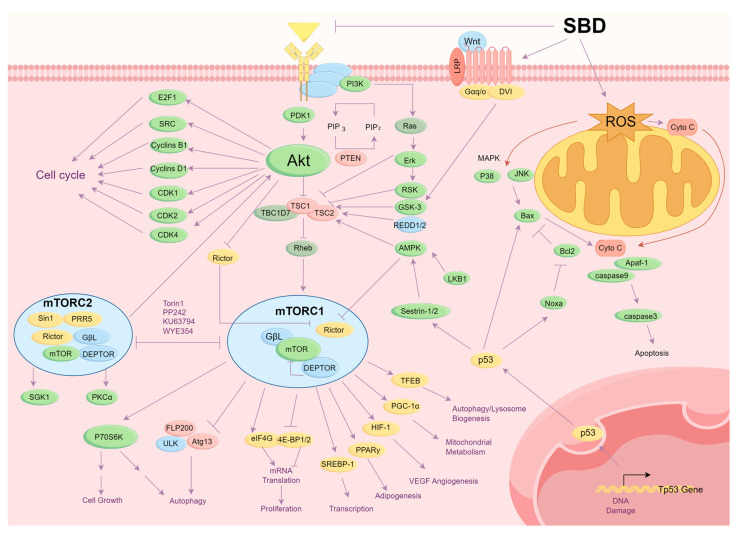
SBD inhibited the proliferation of tumor cells and induced the apoptosis of tumor cells by multiple signaling pathways.

Tumor cells can evade normal cellular regulatory mechanisms, proliferating uncontrollably and invading adjacent tissues. They often produce their own growth factors and activate intracellular signaling pathways such as RAS/Erk and PI3K/Akt/mTOR, which sustain continuous cell proliferation. Therefore, the classical strategy of anti-tumor drugs focuses on inhibiting tumor cell proliferation. As shown in Figure 6, SBD inhibited the proliferation of tumor cells and induced apoptosis of the tumor cells by multiple signaling pathways.

Studies have demonstrated that wogonin and scutellarin in *Scutellaria barbata* can influence the expression of cell-cycle-related proteins by downregulating Cyclins and cyclin-dependent kinases (CDKs), thereby blocking the transition of cells from the G1 to S phase and inhibiting cell proliferation. Hnit et al. found that the combination of *Scutellaria barbata* and *Hedyotis diffusa* extract reduces DNA content accumulation in G2/M phase prostate cancer cells, suggesting inhibition of mitotic regulators and transcriptional suppression, thereby preventing prostate tumor cells from transitioning from the G2 to M phase and inhibiting tumor cell growth. Importantly, this cell cycle arrest is not due to DNA damage but involves the decreased expression of Cyclin B1, CDK1, PLK1, and Aurora A [110]. TP53 is a crucial tumor suppressor gene that inhibits tumor formation by inducing cell cycle arrest or apoptosis. Active ingredients such as quercetin and baicalein in *Hedyotis diffusa–Scutellaria barbata* (HD-SB) upregulate TP53 and P21, promoting cell cycle arrest in HepG2 cells. CDK2, a member of the cyclin-dependent kinase (CDK) family, phosphorylates numerous transcription factors and participates in various cancer signaling pathways, thereby promoting cancer development. TP53 regulates the mRNA and protein expression levels of CDK2 [113]. Li et al. reported that quercetin and luteolin inhibit the activity of CDK2, thereby blocking the progression of hepatocellular carcinoma (HCC) cells through the cell cycle [114]. Furthermore, TP53 enhances the expression of Bcl-2, P21, and P14 while inhibiting BAX expression, promoting apoptosis in liver cancer cells [115]. Sheng et al. treated prostate cancer cells (PCa) with ethanol extracts of *Scutellaria barbata* D. Don (SBD) and found that SBD induces apoptosis and G2/M phase cell cycle arrest in PCa by inhibiting the PI3K/AKT signaling pathway [18]. Su et al. optimized the extraction process of acidic polysaccharides from *Scutellaria barbata* using response surface methodology (RSM), obtaining two highly pure homogeneous acidic polysaccharides SBP-1a and SBP-1b. Both polysaccharides were shown to inhibit the proliferation of HepG2 cells. Additionally, SBP-2A altered the morphology of HepG2 cells, significantly inducing apoptosis by upregulating p53, increasing the Bax/Bcl-2 ratio, and downregulating Cyclin D1 and CDK4, thereby arresting HepG2 cells in the G0/G1 phase of the cell cycle [7].

With the rapid development of interdisciplinary fields such as computer science, life sciences, and medicine, network pharmacology has accelerated drug discovery and development using big data and computational biology techniques. Qi et al. identified quercetin, luteolin, wogonin, and apigenin as the main active flavonoids in *Scutellaria barbata*. Kyoto Encyclopedia of Genes and Genomes (KEGG) analysis indicated that these active ingredients target key pathways such as the glucocorticoid receptor (NR3C1), phosphatidylinositol-4,5-bisphosphate 3-kinase catalytic subunit alpha (PIK3CA), tumor antigen p53 (TP53), transcription factor AP-1 (JUN), mitogen-activated protein kinase 1 (MAPK1), Myc proto-oncogene protein (Myc), cyclin-dependent kinase 1 (CDK1), and ATP-binding cassette transporter ABCG2 (ABCG2) in the treatment of colorectal cancer. GO analysis demonstrated that active ingredients from *Scutellaria barbata* regulate cell cycle arrest to inhibit the proliferation of tumor cells [116]. Yang et al. used the Traditional Chinese Medicines for Systems Pharmacology Database (TCMSP), screened the effective ingredients of *Scutellaria barbata*, and analyzed KEGG and GO to study potential targets for treating liver cancer. The study identified flavonoids such as wogonin, Rhamnazin, quercetin, baicalein, and luteolin, which may act on targets such as CDK1, CDK4, SRC, and E2F1 through pathways including PI3K-Akt signaling, IL-17 signaling, and TNF signaling, thereby inhibiting the proliferation of liver cancer cells [9]. Shi et al. identified some active ingredients including baicalein, wogonin, luteolin, and quercetin from *Scutellaria barbata*, and employed network pharmacology to establish their relationship with nasopharyngeal carcinoma (NPC). Experimental results showed that baicalein and baicalin downregulate key proteins in the PI3K/AKT and p53 signaling pathways in CNE2 cells, inhibiting the proliferation of NPC cells [112].

### 3.2. Induction of Apoptosis in Cancer Cells

Tumor cells typically evade programmed cell death by upregulating anti-apoptotic proteins and downregulating pro-apoptotic proteins. Therefore, inducing apoptosis is a crucial biological mechanism to inhibit cancer progression. Numerous studies have shown that the active components of *Scutellaria barbata* can exert anticancer effects by inducing apoptosis. Research by Bao et al. demonstrated that the active components of *Scutellaria barbata* induce cell apoptosis to inhibit tumor cell growth. As shown in Figure 6, extracts of *Scutellaria barbata* activate the mitochondrial pathway, increasing the Bax/Bcl2 ratio, promoting cytochrome c release, activating downstream Caspase-9 and Caspase-3, and triggering autophagic responses to induce cell apoptosis. Additionally, active components in *Scutellaria barbata* can activate the death receptor pathway, such as the Fas receptor, subsequently activating Caspase-8 and inducing cell apoptosis [16]. Liu et al. found that *Scutellaria barbata* and *Hedyotis diffusa* extracts significantly reduce cell viability in colorectal cancer cells (HCT116) by downregulating Bcl-2, upregulating cleaved-caspase-3 and Bax, promoting ROS generation, and inducing apoptosis. The study also demonstrated significant inhibition of colon cell migration in a dose-dependent manner [17].

Network pharmacology methods have also revealed that the active ingredients of *Scutellaria barbata* can significantly induce cancer cell apoptosis. Zhang et al.’s network pharmacology study suggested that flavonoids such as quercetin, luteolin, and wogonin in SBD downregulate mRNA expression of ovarian-cancer-related genes including AKT1, VEGFA, JUN, TNF, and Caspase-3, inhibiting the PI3K/Akt signaling pathway, thereby inhibiting cancer cell proliferation and inducing apoptosis [111].

Lu et al. constructed a “herb-composition-target-disease” network and predicted that the *Scutellaria barbata* and *Hedyotis diffusa* combination contains 29 flavonoid active components with anti-tumor effects. In vivo and in vitro experiments revealed that these active components significantly induced apoptosis in cancer cells, markedly reduced the expression of p-EGFR, HSP90, p-AKT, p-PI3K, and bcl-2, and increased the expression of ppar, bax, cleaved caspase 3, and cleaved PARP. The results of this study suggest that HD-SB may induce apoptosis by inhibiting the EGFR/ppar/PI3K/AKT pathway [4]. Ma et al. identified 75 components from *Scutellaria barbata* extract using high-performance liquid chromatography and predicted through a “drug-component-target-pathway” model that the *Scutellaria barbata* and *Hedyotis diffusa* combination exerts anti-tumor effects by inhibiting the PI3K/AKT pathway. This was further confirmed by flow cytometry and caspase-3, caspase-8, and caspase-9 activity assays, demonstrating that the *Scutellaria barbata* extract inhibits tumors by inducing apoptosis in cancer cells [117]. Yang et al. isolated the ethyl acetate fraction (EA11) of *Scutellaria barbata* and *Hedyotis diffusa* extract, demonstrating through in vivo and in vitro studies that EA11 reduces the protein expression of PDE7B, PD-L1, β-catenin, and cyclin D1 in 4T1 cells, while increasing cellular cAMP concentration and miR-200c expression. Moreover, EA11 partially exerts its anticancer effects by inactivating the MAPK and AKT signaling pathways. These findings suggest that the ethyl acetate fraction of *Scutellaria barbata* and Hedyotis diffusa extract disrupts the miR-200c-PDE7B/PD-L1-AKT/MAPK axis to prevent breast tumor development [14].

Collectively, these studies demonstrate that flavonoid compounds in *Scutellaria barbata* extract induce cancer cell apoptosis, thereby exerting anticancer effects.

### 3.3. Inhibition of Cell Migration and Invasion

**Figure 7:**
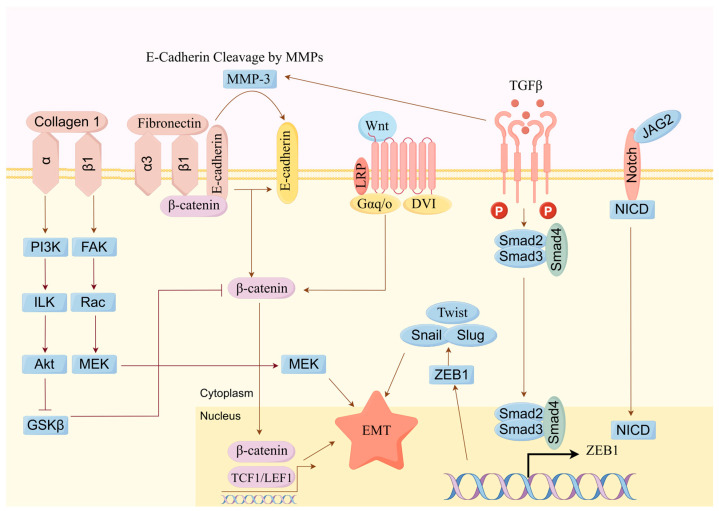
SBD inhibits the invasion and migration of tumor cells through multiple mechanisms.

Cancer cells can metastasize from primary sites through the blood or lymphatic systems, invading healthy tissues to form new tumors, which is a major cause of cancer-related mortality. As shown in Figure 7, studies have shown that the active components of *Scutellaria barbata* can inhibit tumor cell migration and invasion by suppressing the epithelial–mesenchymal transition (EMT) and inhibiting matrix metalloproteinases (MMPs), thereby reducing tumor metastasis.

Zheng et al. evaluated the impact of total flavonoids from *Scutellaria barbata* on breast cancer bone metastasis using in vitro cell experiments and animal models. The results suggested that total flavonoids from *Scutellaria barbata* may reduce the expression of bone-resorbing factor PTHrP and inhibit downstream molecule RANKL/OPG secretion, thereby inhibiting the metastasis of breast cancer cells [12]. Xu et al. found that combined treatment with HD-SB significantly inhibits the proliferation and migration of ovarian cancer cells. Network pharmacology analysis indicated that quercetin, luteolin, and baicalein may be important anticancer components in HD-SB, potentially acting on targets such as EGFR, MAPK1, VEGFA, and PIK3CG to inhibit ovarian cancer growth and migration via focal adhesion pathways [110]. Liu et al. reported that flavonoid components can downregulate the expression and activity of matrix metalloproteinases (MMPs), such as MMP2 and MMP9, thereby inhibiting the invasion and migration ability of tumor cells [118]. Huang et al. discovered that active components of *Scutellaria barbata* can inhibit the phosphorylation of Smad2, Smad3, JNK, p38, and ERK in HepG2 liver cancer cells, blocking TGF-β/Smad/MAPK signaling activation. Concurrently, they observed the downregulation of IntegrinαV, Integrinβ3, MMP2, and MMP9 protein expression, reversing EMT and thereby inhibiting the migration and invasion of liver cancer cells [119].

These studies collectively demonstrate that active components of *Scutellaria barbata* enhance cell adhesion, reduce cancer cell migration and invasion by inhibiting EMT, and suppress MMPs. These processes are mediated by key signaling pathways including TGF-β/Smad/MAPK, EGFR, PI3K/Akt, and RANKL/OPG.

### 3.4. Inhibition of Angiogenesis

Tumor angiogenesis, the formation of new blood vessels that supply nutrients and oxygen to tumors, is crucial for tumor growth and metastasis. Anti-angiogenesis therapy is a key strategy in treating prostate cancer and other cancers. Research conducted by Sheng et al. indicates that *Scutellaria barbata* treatment can inhibit the interaction between PCa cells and HUVECs, thereby suppressing angiogenesis. Additionally, SBD alone or conditioned media from SBD-treated PCa cells reduced HUVEC tube formation on Matrigel without affecting HUVEC viability. Through multiple mechanisms such as inhibiting angiogenesis, inactivating the PI3K/AKT signaling pathway, inducing apoptosis, and arresting the cell cycle at the G2/M phase, *Scutellaria barbata* exhibits strong anti-prostate-cancer activity [18].

Yang et al. found that polysaccharides from *Scutellaria barbata* (PSB) effectively inhibit cell proliferation in vitro and the phosphorylation of human epidermal growth factor receptor (HER) 2. The vivo studies using the Calu-3 subcutaneous xenograft model demonstrated the significant anti-tumor activity of PSB. Immunohistochemistry (IHC) analysis showed PSB dose-dependently reduced microvessel density, indicating its anti-tumor effect is primarily linked to its anti-angiogenesis properties [120]. Shiau et al. investigated how *Scutellaria barbata* regulates the molecular mechanism of hypoxia-inducible factor 1 (HIF-1)-dependent vascular endothelial growth factor (VEGF) expression. The results showed that *Scutellaria barbata* inhibits HIF-1α expression, phosphorylates its upstream signaling mediator AKT, and reduces VEGF expression in tumor cells. Additionally, it inhibits endothelial cell migration and proliferation under hypoxic conditions and significantly inhibits tumor growth in vivo, as evidenced by reducing microvessel density in tumor immunohistochemistry studies [121]. Wei et al. reported that the ethanol extract of *Scutellaria barbata* (EESB) was shown to inhibit tumor growth in colorectal cancer mouse models without affecting body weight gain. Further research revealed that EESB inhibits Sonic Hedgehog (SHH)-mediated tumor angiogenesis, contributing to its anticancer effects [122]. Zhang et al. demonstrated that the active compound luteolin in *Scutellaria barbata* exerts anti-tumor effects through multiple synergistic pathways, including the inhibition of tumor angiogenesis [111]. Similarly, studies by Gong et al. indicate that *Scutellaria barbata* exhibits anti-tumor effects both in vitro and in vivo. The mechanisms through which SBD exerts its anti-tumor effects may include improving the tumor inflammatory microenvironment, blocking the cell cycle, promoting apoptosis, and inhibiting angiogenesis [8]. Additionally, Wang et al. have shown that the primary active components of *Scutellaria barbata*, luteolin, and quercetin, act on core targets such as AKT1, MAPK1, IL6, EGFR, SRC, VEGFA, and TP53. These interactions affect drug resistance, apoptosis, immune regulation, and angiogenesis, contributing to its efficacy against adverse-risk acute myeloid leukemia (AML) [123].

In conclusion, these studies collectively demonstrate that extracts from *Scutellaria barbata* can downregulate vascular endothelial growth factor (VEGF) and its receptor (VEGFR) expression, inhibit the VEGF signaling pathway, suppress angiogenesis, and thereby exert anticancer effects. These mechanisms contribute significantly to the therapeutic potential of *Scutellaria barbata* in cancer treatment strategies.

### 3.5. Reduction of Cancer Cell Drug Resistance

Drug resistance in cancer cells poses a significant challenge in cancer treatment, often leading to decreased efficacy or even treatment failure with many anticancer drugs such as 5-fluorouracil (5-FU) and multidrug resistance (MDR) mechanisms. Combining traditional Chinese medicine with chemotherapy has been shown to mitigate cancer cell resistance and enhance the effectiveness of chemotherapy.

Research by Lin et al. demonstrated that EESB reduces the expression of CyclinD1, Bcl-2, and ATP binding cassette subfamily G member 2 (ABCG2), while upregulating p21 and Bax expression in HCT-8/5-FU colorectal cancer cells. This modulation suppresses the activation of the PI3K/AKT pathway, thereby inhibiting chemotherapy resistance in these cells [124]. Shao et al. found that salvigenin, an active compound from *Scutellaria barbata*, enhances the sensitivity of hepatocellular carcinoma (HCC) cells to 5-FU and attenuates the resistance of HCC 5-FU-resistant cells to the drug. Salvigenin achieves its anticancer effects by inhibiting the PI3K/AKT/GSK-3β pathway, thereby impeding aerobic glycolysis and 5-FU chemotherapy resistance in HCC cells [10]. Li et al., using modern isolation and purification techniques, isolated and identified novel clerodane-type diterpenoid compounds from *Scutellaria barbata*. The vitro evaluation of these compounds revealed their ability to reverse multidrug resistance in HepG2/Adr cells when combined with doxorubicin (Adr). Experimental evidence suggested that these diterpenoid compounds may reverse tumor cell multidrug resistance by inhibiting the P-glycoprotein efflux function [125]. Zhang et al. reported that the anticancer effects of *Scutellaria barbata* may involve multiple compounds acting on various targets and pathways in ovarian cancer. For instance, wogonin enhances apoptosis in ovarian cancer CAOV3/DDP cells resistant to cisplatin by reducing Bcl-2 expression, thereby augmenting cisplatin’s anti-tumor effects. Additionally, wogonin treatment increases tumor cell apoptosis and enhances the cytotoxicity of TNF-α and TRAIL to tumor cells, blocks the tumor cell cycle, inhibits tumor angiogenesis, and synergizes with chemotherapy drugs through ROS and Ca^2+^-mediated signaling pathways [111]. Wang et al. showed that luteolin and quercetin were the main active components of *Scutellaria barbata* against adverse-risk acute myeloid leukemia (AML) through the network pharmacology research. These compounds were found to affect drug resistance, cellular behaviors, apoptosis, immune regulation, cell metabolism, angiogenesis, and other signaling pathways through core targets such as AKT1, MAPK1, IL6, EGFR, SRC, VEGFA, and TP53 [123].

*Scutellaria barbata* exerts its potential in overcoming cancer drug resistance by mechanisms such as inhibiting drug efflux pumps and controlling apoptosis, thereby enhancing the efficacy of anticancer drugs. Future research aims to elucidate its precise mechanisms of action and explore its clinical application value in anticancer therapy.

### 3.6. Improvement of Tumor Microenvironment

The tumor microenvironment (TME) refers to the surroundings in which tumor cells exist, comprising blood vessels, immune cells, fibroblasts, bone-marrow-derived inflammatory cells, various signaling molecules, and extracellular matrix. This microenvironment plays a crucial role in the initiation, growth, and metastasis of tumors through continuous interactions. Tumors can influence their microenvironment by releasing signaling molecules to promote angiogenesis and induce immune tolerance. Conversely, immune cells within the microenvironment can also affect the growth and development of cancer cells.

Gong et al. reported that *Scutellaria barbata* inhibits the proliferation of HepG2 cells in vitro in a dose-dependent manner and significantly suppresses tumor growth in mice. *Scutellaria barbata* can modulate the infiltration of Treg and Th17 cells in the tumor microenvironment, reducing the number of CD4^+^,CD25^+^,Foxp3^+^,Treg cells and Th17 cells in tumor tissues. This modulation decreases IL-10, TGF-β, and IL-17A levels in the serum of liver cancer mice (*p* < 0.01), while increasing IL-2 and IFN-γ levels (*p* < 0.01) [8]. Li et al. showed that a 1:1 mixture of *Scutellaria barbata* and *Scutellaria barbata* ethyl acetate extracts significantly downregulates LMO1 expression. LMO1 overexpression weakens the inhibitory effects of *Scutellaria barbata* on cell proliferation and invasion, inducing an inflammatory tumor microenvironment. Additionally, downregulating LMO1 expression can suppress cell proliferation and migration by reducing AKT/mTOR pathway activation [13]. Zhang et al. showed that AKT1, VEGFA, JUN, TNF, and Caspase-3 are central among all target genes through protein–protein interaction network analysis. VEGFA is often overexpressed in solid tumors and malignant diseases, significantly promoting tumor angiogenesis. Cell experiments demonstrated that active compounds such as baicalein and wogonin from *Scutellaria barbata* inhibit VEGFA expression. This targeted regulation affects other cell types within the tumor microenvironment and influences tumor function [111].

## 4. Clinical Application

In the clinical practice of Traditional Chinese Medicine (TCM), several compound formulas containing *Scutellaria barbata* are utilized for the treatment or adjunctive therapy of tumors. For instance, “Intestine Formula” is used for advanced metastatic colorectal cancer, Compound Banmao Capsules combined with cisplatin and paclitaxel injection (DP) are employed for advanced ovarian cancer, Yipi Fuzheng Recipe effectively reduces the incidence of chemotherapy-induced nausea and vomiting in esophageal cancer patients, Yangzheng Xiaojie Capsules improve the quality of life and prolong life expectancy in patients with advanced gastrointestinal cancers, Ankangxin Capsules are applicable for the treatment of lung cancer, cervical cancer, liver cancer, and gastric cancer, Anticancer Pills are used for HER2-positive advanced gastric cancer, Ban zhi Qin lian decoction is prescribed for non-small-cell lung cancer, and *Hedyotis diffusa–Scutellaria barbata* is used for maintenance therapy in malignant tumors. This section reviews the clinical studies on *Scutellaria barbata*, providing a straightforward assessment of its therapeutic effects [126,127,128,129,130,131,132,133].

A clinical study targeting advanced refractory colorectal cancer patients who had progressed after multiple chemotherapy regimens randomized them into treatment and control groups, each comprising 30 cases. The treatment group received Intestine Formula (containing *Scutellaria barbata*) along with optimal supportive care, while the control group received only optimal supportive care until patient loss to follow-up or death. The results showed that after 2 months, the treatment group demonstrated an improvement rate in TCM symptom scores of 76.7%, a total effective rate by Karnofsky Performance Score of 86.7%, and a tumor control rate of 60.0%, all significantly higher than the control group’s rates of 43.3%, 53.3%, and 33.3%, respectively (*p* < 0.05). Immunologically, the treatment group showed a significant increase in NK cell count and CD4/CD8 ratio compared to before treatment (*p* < 0.05), effectively improving patient symptoms and modulating immunity. The treatment group’s half-year survival rate, one-year survival rate, and two-year survival rate were 92.8%, 57.1%, and 14.2%, respectively, with a median survival period of 13 months, all significantly higher than those of the control group (*p* < 0.05) [126].

A clinical study investigating the clinical efficacy of Compound Banmao Capsules combined with the DP regimen (cisplatin plus paclitaxel injection) in treating advanced ovarian cancer included 114 patients receiving treatment. Using randomization, patients were divided into treatment and control groups, with 57 cases in each. The control group received the DP chemotherapy regimen, while the treatment group received oral Compound Banmao Capsules (containing *Scutellaria barbata*) in addition to the control group’s treatment. The results indicated that post-treatment, the disease control rates were 45.6% and 68.4% for the control and treatment groups, respectively, and the objective response rates were 63.2% and 77.2%, respectively, with statistically significant differences (*p* < 0.05). One-year survival rate, three-year survival rate, progression-free survival (PFS), and overall survival (OS) were significantly higher in the treatment group compared to the control group, with statistically significant differences observed between the two groups (*p* < 0.05) [127].

An observational clinical study assessing the clinical efficacy of Yipi Fuzheng Formula combined with chemotherapy in treating advanced esophageal cancer included 41 patients. Using random numerical methods, the patients were allocated into a treatment group *(n* = 20) and a control group (*n* = 21). The control group received paclitaxel plus nedaplatin chemotherapy regimen, while the treatment group received Yipi Fuzheng Formula (containing *Scutellaria barbata*) in addition to the control group’s treatment. The results demonstrated statistically significant differences between the treatment and control groups in terms of improvement in TCM syndrome scores, TCM syndrome effectiveness, Karnofsky Performance Score (KPS), and immune indicators (*p* < 0.05). The treatment group showed improvements in nausea and vomiting symptoms (*p* < 0.05) and reduced the incidence of side effects [128] to some extent.

An observational clinical study on the combined use of Endu (Enzalutamide) and Yangzheng Xiaoji Capsules in treating advanced gastrointestinal malignant tumors included 80 patients. They were randomly divided into a control group of 38 cases and an observation group of 42 cases. The control group received Endu combined with capecitabine maintenance therapy, while the observation group received Endu combined with Yangzheng Xiaoji Capsules. The results showed that after treatment, both groups exhibited significant improvements in serum inflammatory factors TNF-α, IL-6, and CRP compared to before treatment (*p* < 0.05). CD4^+^ and CD4^+^/CD8^+^ ratios increased significantly post-treatment (*p* < 0.05), with more pronounced changes in the observation group compared to the control group (*p* < 0.05). Changes in KPS scores were more significant in the observation group than the control group (*p* < 0.05). The observation group showed significantly better scores in functional domains, symptom domains, and overall survival quality compared to the control group (*p* < 0.05). There were no statistically significant differences in one-year PFS rates and OS rates between the two groups (*p* > 0.05), and adverse reactions were significantly fewer in the observation group (*p* > 0.05) [129].

A clinical study exploring the application effectiveness of Ankangxin Capsules combined with cisplatin in adjuvant chemotherapy for non-small-cell lung cancer (NSCLC) involved 90 patients. They were randomly divided into a control group and an observation group, each comprising 45 cases. The control group received a gemcitabine plus cisplatin (GP) regimen, while the observation group received Ankangxin Capsules in addition to the control group’s treatment. The results showed that the clinical benefit rate in the observation group was 66.67%, significantly higher than the control group’s 46.67%, with statistically significant differences (*p* < 0.05). CD3^+^, CD4^+^, CD8^+^, CD4^+^/CD8^+^, and NK levels were significantly higher in the observation group compared to the control group (*p* < 0.05). The observation group had significantly lower rates of nausea, vomiting, rash, neutropenia, thrombocytopenia, anemia, hepatic dysfunction, and toxic side effects compared to the control group (*p* < 0.05) [130]. 

A clinical study investigating the short-term and long-term efficacy of Anticancer Pills combined with trastuzumab and chemotherapy in HER2-positive advanced gastric cancer patients included 98 subjects. They were randomly divided into an observation group and a control group, each comprising 49 cases. The control group received a trastuzumab and mFOLFOX6 chemotherapy regimen, while the observation group additionally received Anticancer Pills orally. After treatment, the observation group showed significantly higher effective rates and control rates compared to the control group (*p* < 0.05). PFS, OS, one-year survival rate, and three-year survival rate were all significantly higher in the observation group compared to the control group (*p* < 0.05). During treatment, the observation group had significantly lower incidence rates of cardiac toxicity, gastrointestinal reactions, hepatic toxicity, renal toxicity, bone marrow suppression, and neurological toxicity compared to the control group (*p* < 0.05) [131].

A clinical efficacy analysis study on the combination of Ban zhi Qin lian decoction and gefitinib in treating elderly patients with NSCLC included 63 elderly NSCLC patients. According to the treatment nature, they were randomly divided into a gefitinib treatment group (control group) with 31 cases and a Ban zhi Qin lian decoction combined with gefitinib treatment group (study group) with 32 cases. The results showed that the study group achieved a 100% probability of disease remission and stabilization, significantly higher than the control group’s 81.3% (*p* < 0.05), effectively alleviating clinical discomfort symptoms and improving patient comfort comprehensively [132].

A clinical study on the maintenance treatment effectiveness of *Hedyotis diffusa–Scutellaria barbata* in malignant tumor maintenance treatment selected 80 gastric cancer patients who completed first-line effective chemotherapy. Using a double-blind principle, the patients were divided into a control group and a maintenance treatment group, each with 40 cases. The control group received routine maintenance treatment, while the maintenance treatment group received *Hedyotis diffusa–Scutellaria barbata* for maintenance treatment in addition to the control group’s treatment. The results indicated that the maintenance treatment group had a significantly higher effective rate (RR) than the control group, with statistically significant differences (*p* < 0.05). The maintenance treatment group also showed significantly higher improvement rates in overall quality of life compared to the control group (*p* < 0.05), and the incidence of adverse reactions in the maintenance treatment group was significantly lower than that in the control group (*p* < 0.05) [133].

## 5. Conclusions and Future Prospects

*Scutellaria barbata*, a traditional Chinese medicinal herb, has a medicinal history spanning over four centuries. This plant contains a wealth of chemical constituents, primarily including flavonoids, diterpenoids, polysaccharides, and essential oils. These components bestow *Scutellaria barbata* with broad pharmacological activities, particularly demonstrating significant potential in the field of anti-tumor therapy.

This paper comprehensively summarized the research findings on *Scutellaria barbata* from the past decade, identifying 72 flavonoids and 164 diterpenoids. The main active flavonoid constituents include quercetin, luteolin, scutellarein, scutellarin, wogonin, and baicalein, which are pivotal to the anti-tumor effects of *Scutellaria barbata*. Diterpenoids are more numerous compared to other components due to their diverse core structures and abundant substituents. Studies indicate that diterpenoids possess certain cytotoxic and anti-tumor activities.

Modern pharmacological studies have gradually elucidated the anti-tumor mechanisms of *Scutellaria barbata*, including the inhibition of tumor cell proliferation, promotion of tumor cell apoptosis, inhibition of cell invasion and migration, regulation of the immune system, and anti-angiogenesis. The PI3K/Akt signaling pathway is abnormally activated in tumor cells. The activation promotes cancer cell proliferation, survival, migration, and invasion while inhibiting apoptosis. According to the literature, *Scutellaria barbata* extract can inhibit the phosphorylation of PI3K and Akt. On one hand, the inhibition of the PI3K/Akt signaling pathway can suppress proto-oncogenes and reduce the expression of Src Family Kinases and TNF genes, thereby inhibiting tumor cell proliferation and migration and promoting apoptosis. On the other hand, it can reduce the expression of cell cycle proteins such as Cyclins B1, Cyclins D1, CDK1, CDK2, and CDK4, preventing the transition of tumor cells from the G2 phase to the M phase and inhibiting cell proliferation. SBD can inhibit the expression of NFκB in tumor cells, thereby activating the tumor suppressor gene TP53 and promoting the expression of tumor necrosis factor Fas. The overexpression of TP53 also affects cell cycle proteins like CDK2, causing cell cycle arrest. Meanwhile, TP53 and Fas induce the downregulation of the anti-apoptotic factor Bcl-2 and the upregulation of the pro-apoptotic protein Bax. The increasing Bax/Bcl-2 ratio also enhances the expression of cytochrome C. Cytochrome C can not only directly disrupt the mitochondrial membrane, inducing cancer cell apoptosis, but also can form apoptosomes with Caspase9 and Apaf-1, activating the expression of Caspase3 and inducing cancer cell apoptosis. Research has also reported that SBD can induce the production of ROS, mediated by the MAPK pathway, leading to the expression of pro-apoptotic factors such as JNK and P38, thereby triggering cancer cell apoptosis. *Scutellaria barbata* can also inhibit EMT through the TGF-β and Wnt/β-catenin pathways, preventing cancer cell invasion and migration. SBD inhibits tumor angiogenesis by suppressing the VEGF pathway, exerting an anticancer effect. Extracts of *Scutellaria barbata* inhibit the activation of the PI3K/Akt pathway, reducing the expression level of the ABC transporter ABCG2, suppressing the efflux function of ABC transporters, and increasing drug accumulation in cancer cells, thereby exhibiting anti-tumor activity. In terms of the anti-tumor mechanisms of *Scutellaria barbata* extracts, although sufficient evidence exists, a considerable portion of research has been conducted through network pharmacology combined with simple in vitro experiments, failing to validate related mechanisms in vivo. Future studies on the anti-tumor mechanisms of *Scutellaria barbata* extract require further in-depth investigations to support its clinical anti-tumor applications.

Clinically, *Scutellaria barbata* has been widely used in the treatment of various tumors such as prostate cancer, ovarian cancer, gastrointestinal cancer, and lung cancer. In adjuvant chemotherapy and radiotherapy, *Scutellaria barbata* has been shown to improve patient symptoms, reduce adverse reactions to chemotherapy, enhance quality of life, and increase therapeutic efficacy. However, the clinical application of *Scutellaria barbata* still faces challenges such as the standardization of components, determination of dosage, and long-term safety assessment.

Despite some progress in this research, there remain many unknown aspects of the chemical constituents and anti-tumor mechanisms. Firstly, identifying, isolating, and elucidating the specific molecular structures for the observed anti-tumor effects is a meticulous and time-consuming process. Moreover, the anti-tumor mechanisms of these compounds are often multifaceted. The intricacies of these interactions, often involving intricate networks of proteins, genes, and metabolic pathways, render them difficult to fully comprehend and harness for therapeutic purposes. Furthermore, the variability in tumor biology across different cancer types and even within individual patients presents another layer of complexity. This underscores the need for a more personalized approach to cancer treatment, where the specific chemical constituents and mechanisms of action are tailored to the unique characteristics of each patient’s tumor. In conclusion, despite the progress made in recent years, the chemical constituents and anti-tumor mechanisms of many potential cancer therapeutics remain largely unknown. Advances in technology and ongoing research efforts are critical to unraveling these mysteries and developing more effective and targeted cancer treatments.

## Figures and Tables

**Table 1:** Flavonoids in *Scutellaria barbata*.

No.	Compound	R5	R6	R7	R8	R2’	R3’	R4’	R5’	R6’	Ref.
Flavones											
**1**	luteolin	OH	H	OH	H	H	H	OH	OH	H	[21]
**2**	apigenin	OH	H	OH	H	H	H	OH	H	H	[22,23]
**3**	scutellarein	OH	OH	OH	H	H	H	OH	H	H	[21]
**4**	wogonin	OH	H	OH	OMe	H	H	H	H	H	[24]
**5**	6-*O*-methylscutellarein	OH	OMe	OH	H	H	H	OH	H	H	[25]
**6**	4′,5-dihydroxy-3′,5′,6,7-tetramethoxyflavone	OH	OMe	OMe	H	H	OMe	OH	OMe	H	[26,27]
**7**	5-hydroxy-7,8-dimethoxyflavone	OH	H	OMe	OMe	H	H	H	H	H	[28]
**8**	rivularin	OH	H	OMe	OMe	OMe	H	H	H	OH	[28]
**9**	4′-hydroxy-wogonin	OH	H	OH	OMe	H	H	OH	H	H	[27,29]
**10**	5,7,3′,4′,5′-pentamethoxyflavone	OMe	H	OMe	H	H	OMe	OMe	OMe	H	[30]
**11**	5-hydroxy-7,3′,4′,5′-tetramethoxyflavone	OH	H	OMe	H	H	OMe	OMe	OMe	H	[30]
**12**	5-hydroxy-7,4′-dimethoxy-flavone	OH	H	OMe	H	H	H	OMe	H	H	[31]
**13**	5-hydroxy-7,8,4′-trimethoxyflavone	OH	H	OMe	OMe	H	H	OMe	H	H	[31]
**14**	baicalein	OH	OH	OH	H	H	H	H	H	H	[32]
**15**	isoscutellarein	OH	H	OH	OH	H	H	OH	H	H	[33]
**16**	6-hydroxyluteolin	OH	OH	OH	H	H	H	OH	OH	H	[33]
**17**	5-hydroxy-6,7,3′,4′-tetramethoxyflavone	OH	OMe	OMe	H	H	H	H	H	H	[33]
**18**	5,6,7,3′,4′,5′-hexahydroxyflavone	OH	OH	OH	H	H	OH	OH	OH	H	[34]
**19**	6-methoxyluteolin	OH	OMe	OH	H	H	H	OH	OH	H	[34]
**20**	chrysoeriol	OH	H	OH	H	H	H	OH	OMe	H	[34]
**21**	cirsiliol	OH	OMe	OMe	H	H	OH	OH	H	H	[34]
**22**	tenaxin	OH	OMe	OMe	OMe	H	H	H	H	OH	[34]
**23**	wogonoside	OH	H	OGlcA	H	H	H	H	H	H	[34]
**24**	apigenin-5-*O*-β-d-glucopyranoside	Glu	H	OH	H	H	H	OH	H	H	[35]
**25**	apigenin-7-*O*-β-d-glucoside	OH	H	O-Glu	H	H	H	OH	H	H	[36]
**26**	apigenin-7-*O*-neohesperidoside	OH	H	O-Neo	H	H	H	OH	H	H	[37]
**27**	hyperoside	Refer to Figure 2									[30]
**28**	baicalin	OH	OH	O-GlcA	H	H	H	H	H	H	[24]
**29**	salvigenin	OH	OMe	OMe	H	H	H	OMe	H	H	[31]
**30**	luteolin-7-*O*-β-d-glucopyranoside	OH	H	O-Glu	H	H	OH	OH	H	H	[36]
**31**	scutellarein-7-*O*-glucoside	OH	OH	O-Glu	H	H	H	H	H	H	[34]
**32**	isoscutellarein-8-*O*-β-d-glucuronide-6″-methylester	OH	H	OH	Glu-Me ester	H	H	OH	H	H	[33]
**33**	apigenin-7-*O*-β-d-glucuronide-6″-methylester	OH	H	O-GlcA-Me ester	H	H	H	OH	H	H	[33]
**34**	5-hydroxy-4′-methoxyflavone-7-*O*-*α*-Lrhamnosyl-(1→6)-β-d-glucopyranoside	OH	H	O-Rha-Glu	H	H	H	OMe	H	H	[38]
**35**	scutellarin	OH	OH	O-GlcA	H	H	H	OMe	H	H	[39]
**36**	5,8,2′-trihydroxy-7-*O*-flavonoid glucuronide	OH	H	O-GlcA	OH	OH	H	H	H	H	[40]
**37**	apigenin-7-*O*-β-d-glucuronide	OH	H	O-GlcA	H	H	H	OH	H	H	[37]
**38**	kaempferol-3-*O*-β-d-rutinoside	Refer to Figure 2									[41]
**39**	isoscutellarein-8-*O*-glucuronide	OH	H	OH	O-GlcA	H	H	OH	H	H	[33]
**40**	scutellarein-7-*O*-glucoside	OH	OH	O-Glu	H	H	H	OH	H	H	[34]
**41**	luteolin-7-O-glucuronide	OH	H	O-GlcA	H	H	OH	OH	H	H	[34]
**42**	isoscutellarein-7-*O*-glucuronide	OH	H	O-GlcA	OH	H	H	OH	H	H	[34]
**43**	hispidulin-7-*O*-β-d-methylgluzcuronide	OH	OMe	O-GlcA	H	H	H	OH	H	H	[33]
**44**	scutellarein-7-*O*-β-d-glucuronide methyl ester	OH	OH	O-GlcA Me ester	H	H	H	OH	H	H	[33]
**45**	4-hydroxy-wogonin-7-*O*-glucuronide	OH	H	O-GlcA	OMe	H	H	OH	H	H	[34]
**46**	8-hydroxyluteolin-7-*O*-glucuronide	OH	H	O-GlcA	OH	H	OH	OH	H	H	[34]
**47**	6-hydroxyluteolin-7-*O*-glucuronide	OH	OH	O-GlcA	H	H	OH	OH	H	H	[34]
Flavonols											
**48**	quercetin	OH	H	OH	H	H	OH	OH	OH	H	[39]
**49**	isorhamnetin	OH	H	OH	H	H	H	OH	OMe	H	[32]
**50**	quercetin-4′-*O*-glucuronide	OH	H	OH	H	H	OH	GlcA	H	H	[39]
Flavonones											
**51**	carthamidin	OH	OH	OH	H	H	H	OH	H	H	[35]
**52**	naringenin	OH	H	OH	H	H	H	OH	H	H	[25]
**53**	6-methoxynaringenin	OH	OMe	OH	H	H	H	OH	H	H	[25]
**54**	5,7,4′-trihydroxy-8-methoxyflavanone	OH	H	OH	OMe	H	H	OH	H	H	[25]
**55**	dihydrooroxylin A	OH	OMe	OH	H	H	H	H	H	H	[25]
**56**	isocarthamidin	OH	H	OH	OH	H	H	OH	H	H	[25]
**57**	eriodictyol	OH	H	OH	H	H	OH	OH	H	H	[27]
**58**	naringenin-4′-*O*-glucuronide	OH	H	OH	H	H	H	O-GlcA	H	H	[34]
**59**	8-methoxynaringenin-7-*O*-glucuronide	OH	H	O-GlcA	OMe	H	H	OH	H	H	[34]
**60**	naringenin-7-*O*-glucuronide	OH	H	O-GlcA	H	H	H	OH	H	H	[34]
**61**	6-methoxynaringenin-7-*O*-glucuronide	OH	OMe	O-GluA	H	H	H	OH	H	H	[34]
**62**	isocarthamidin-7-*O*-glucuronide	OH	H	O-GluA	OH	H	H	OH	H	H	[34]
**63**	carthamidin-7-*O*-glucuronide	OH	OH	O-GluA	H	H	H	OH	H	H	[34]
**64**	5,6,7,3′,4′-pentahydroxyflavanone-3-*O*-glucuronide	OH	OH	O-GlcA	H	H	OH	OH	H	H	[34]
Flavanonols											
**65**	7-hydroxy-2′,5,8-trimethoxyflavanone	OMe	H	OH	OMe	OMe	H	H	H	H	[27]
**66**	2(*S*)-2′,7-dihydroxy-5,8-dimethoxyflavanone	OMe	H	OH	OMe	OH	H	H	H	H	[35]
**67**	3,5,6,7,4′-pentahydroxyflavanone	OH	H	H	OH	H	OH	OH	H	H	[34]
**68**	3,5,7,8,4′-pentahydroxyflavanone)	OH	H	OH	OH	H	H	OH	H	H	[34]
**69**	3,5,7,4′-tetrahydroxy-6-methoxyflavanone	OH	OMe	OH	H	H	H	OH	H	H	[34]
**70**	3,5,7,4′-tetrahydroxy-8-methoxyflavanone)	OH	H	OH	OMe	H	H	OH	H	H	[34]
**71**	3,5,8,3′,4′-pentahydroxyflavanone-3-*O*-glucuronide	OH	H	O-GlcA	OH	H	OH	OH	H	H	[34]
Chalcones											
**72**	2′,4′-dihydroxy-2,3′,6′-trimethoxy-chalcone	Refer to Figure 2									[35]

**Table 2:** The neo-clerodane-type diterpenoids isolated from *Scutellaria barbata*.

No.	Compound	R1	R2	R3	Reference
Type A					
**73**	scutebata X	Nic	Bz	O	[32,42,43,44]
**74**	scutebata Y	Nic	Bz	β-OH, H	[43]
**75**	2-carbonylscutebartatin A	Nic	Nic	O	[45]
**76**	scutebata I	Ac	H		[42,46]
**77**	scutebata J	Bz	H		[47,48]
**78**	scutebata K	Su	H		[42,43,46,47]
**79**	scutebata T	Ac	De		[44,48]
**80**	barbatin C	H	H		[42,49,50]
**81**	barbatin D	Bz	Bz		[43,48,51]
**82**	barbatin E	Hs	Ht		[51]
**83**	barbatin F	Ht	Bz		[52]
**84**	6-*O*-nicotinoylbarbatin A	Nic	H		[45,53]
**85**	6, 7-di-*O*-acetoxybarbatin A	Ac	Ac		[45,49]
**86**	scutebarbatine A	Nic	Nic		[48,54,55,56]
**87**	scutebarbatine B	Nic	Bz		[50,55]
**88**	scutebarbatine K	Nic	Ac		[48,57]
**89**	scutebarbatine L	Nic	Hs		[43,48,55,57]
**90**	scutebarbatine Y	Bz	Nic		[32,48,54]
**91**	scutebartine F	Nic	Bz	Z(R_Δ_^11–12^)	[42,55]
**92**	scutebartine G	Nic	Nic	Z(R_Δ_^11–12^)	[42,55]
**93**	scutolide A	Ac	De	Z(R_Δ_^11–12^)	[44,48]
**94**	scutolide C	Bz	De		[48]
**95**	scutolide D	Bz	Hs		[48]
**96**	scutolide E	Ac	Bz		[48]
**97**	scutehenanine A	H	Nic		[42,53]
**98**	6-*O*-(2-carbonyl-3-methylbutanoyl)-scutehenanine A	Ht	Nic		[53]
**99**	6-*O*-acetylScutehenanine A	Ac	Nic		[42]
**100**	scutelinquanine C	Nic	Ht		[58]
**101**	barbaolide M	Ht	Mepr		[59]
**102**	scutebarbatolide A	Bz	Ac		[60]
**103**	scutebarbolide J	Su	OH		[42]
**104**	scutebarbolide K	OH	Su		[42]
**105**	8-*O*-nicotinoylbarbatin A				[45]
**106**	6-(2, 3-epoxy-2isopropyl-n-propoxyl) -barbatin C				[61]
**107**	scutolide B				[42,48,55]
**108**	scutebarbolide I				[42]
Type B					
**109**	scutebata L	Bz	Bz	H	[46,47]
**110**	scutebarbatine D	Nic	Bz	OH	[62]
**111**	scutebarbatine O	Nic	Nic	OH	[63]
**112**	scutehenanine A	Bz	Bz	OH	[42,53]
**113**	scutebarbatine E	Nic	Bz	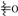	[62]
**114**	scutellone D	OH			[64,65,66]
**115**	scutellone E				[66]
**116**	scutellone F				[67,68]
**117**	scutellone H	OEt			[68,69]
**118**	scutellone I	OMe			[68]
**119**	scutebarbatine C				[62]
Type C					
**120**	scutebata A	Bz	Bz	OH	[70,71]
**121**	scutebata B	Nic	Bz	OH	[25,54,70]
**122**	scutebata C	Nic	H	OH	[54,70]
**123**	scutebata W	Su	H	H	[42,71]
**124**	barbatin G	H	Nic	OH	[52]
**125**	scutebarbatine X	Nic	Nic	OH	[32,49,54,55]
**126**	scutebarbolide D	Bz	Nic	OH	[25,32,42]
**127**	scutebarbolide E	Bz	De	OH	[42]
**128**	scutolide F	Bz	H	H	[48]
**129**	scutolide G	Ac	De	H	[48]
**130**	scutolide H	Nic	De	H	[48]
**131**	scutolide I	Ac	Ac	H	[48]
**132**	scutolide J	Nic	Nic	H	[48]
**133**	barbatellarine B	Nic	Bz	H	[48,72]
**134**	scutebata M	Nic	Hs	OH	[46,55,72]
**135**	scutebarbatine Z	Nic	H	H	[32,55]
**136**	6-acetoxybarbatin C	Ac	H	OH	[73]
**137**	scutebarbolide A	Ac	H		[42]
**138**	scutebarbolide C	H	OH		[42]
**139**	scutebarbolide B				[42]
**140**	scutebarbolide F				[42]
**141**	scutebarbolide G				[42]
**142**	scutebarbolide H				[42]
**143**	barbaolide J	Nic	H	OH	[59]
**144**	barbaolide K				[59]
**145**	barbaolide L	Nic	Ac	H	[59]
**146**	scutebarbatolide B	Nic			[60]
**147**	scutebarbatolide C	Bz			[60]
**148**	scuttenline A	Nic	OB		[74]
**149**	scuttenline B				[74]
**150**	scuttenline C	Bz	H		[74]
**151**	scuttenline D				[74]
Type D					
**152**	scutebata P	Bz	H	OBz(13*S*)	[49,70,75]
**153**	scutebata Q	Bz	OBz	H(13*R*)	[43,75]
**154**	scutehenanine C	Nic	H	Nic	[53]
**155**	scutebata N	Bz	H	Bz	[46,47]
**156**	barbatin B				[50]
**157**	barbatellarine C				[74]
**158**	barbatellarine D				[74]
**159**	barbatellarine E				[74]
**160**	barbatellarine F				[72,76]
**161**	scuterivulactone C1				[64,65]
**162**	scuterivulactone C2				[64,65]
**163**	scutellone C				[67,68]
**164**	scutellone G				[68,69]
**165**	barbatellarine A				[72]
Type E					
**166**	scutebata D	Ac	Ac	Bz	[44,48,54,71]
**167**	scutebata E	Ac	Ac	MePr	[49,70,71]
**168**	scutebata F	Ac	Ac	Nic	[48,49,71]
**169**	scutebata G	Nic	Bz	Bz	[43,54,71]
**170**	scutebata O	Bz	H	H	[42,46,47]
**171**	scutebata R	Ac	Ac	De	[43,75]
**172**	scutebata S	Ac	De	Bz	[44]
**173**	scutebata V	Nic	Nic	H	[46]
**174**	scutebata B1	Ac	Nic	Nic	[43]
**175**	scutebata C1	Bz	H	Bz	[43]
**176**	scutebartine A	Nic	H	Bz	[55]
**177**	scubatine E	Ac	Bz	Bz	[70]
**178**	scubatine F	Bz	Ac	Bz	[70]
**179**	barbatine C	Ac	Ac	Nic	[42,55]
**180**	barbatine D	Nic	Ac	Nic	[55,71]
**181**	scutebatin A	Nic	Bz	Nic	[43,54,63]
**182**	scutebatin B	Nic	Nic	Bz	[54,63]
**183**	barbatin H	Ac	Nic	Nic	[48]
**184**	scutebarbatine W	Nic	H	OBz	[32,54,55]
**185**	scutebartine B	Ac	Ac	Nic	[55]
**186**	scutebatin C	Nic	H	Nic	[54]
**187**	scutebarbolide L	Ac	Ac	Ac	[42]
**188**	scutebartine C	Nic	Nic	H	[42,55]
**189**	scutebartine D	Nic	Nic	OH	[55]
**190**	scutolide K	Bz	Bz	H	[48,70]
**191**	scutolide L	Bz	Bz	OH	[48]
**192**	scutelinquanine D	H	Nic	H	[73]
**193**	scubatine D	Ac	Ac	Ac	[70]
**194**	barbatine A	Nic	Ac	Nic	[42,55,77]
**195**	barbatine B	Nic	Nic	Nic	[48,77]
**196**	barbatin A	Bz	H	Bz	[32,50]
**197**	scutebarbatine F	Nic	Ac	Ac	[32,54]
**198**	scutebarbatine G	H	H	Nic	[32,54,78]
**199**	6-*O*-nicotinoyl-7-*O*-acetylscutebarbatine G	Nic	Ac	Nic	[32,78]
**200**	6-*O*-nicotinoylscutebarbatine G	Nic	H	Nic	[32,54,55]
**201**	6,7-di-*O*-nicotinoylscutebarbatine G	Nic	Nic	Nic	[32,78]
**202**	scutehenanine B	Nic	H	Nic	[32,56]
**203**	scutebarbolide M	Ac	Ac	Ac	[42]
**204**	scutebata Z	Ac	Nic	H(13*S*)	[43,55]
**205**	scutebata A1	Ac	Nic	H(13*R*)	[43]
**206**	scutebartine E				[55]
**207**	scutebata U				[46]
**208**	scutehenanine H				[61]
**209**	barbaolide A	H	Nic	Ac	[59]
**210**	barbaolide B	ONic	Nic	MePr	[59]
**211**	barbaolide C	ONic	Ac	MePr	[59]
**212**	barbaolide D				[59]
**213**	barbaolide E	H	Ac	MePr	[59]
**214**	barbaolide F	Ac	MePr	H	[59]
**215**	barbaolide G				[59]
**216**	barbaolide H	Bz	Ac	OH	[59]
**217**	barbaolide I	Ac	Ac	ONic	[59]
**218**	scutellone J	Ac	H	Ac	[79]
**219**	scutelinquanine A	Ac	H	Nic	[58]
Type F					
**220**	scutebata H	OH	Bz	Ac	[45,46]
**221**	scutebartine H	OMe	H	Nic	[55]
**222**	scutebartine I	H	OMe	Nic	[55]
**223**	scutebartine J	OH	Nic	Ac	[55]
**224**	scutebarbatine I	H	OEt		[57]
**225**	scutebarbatine J	OEt	H		[57]
**226**	scutellin A				[80]
Type G					
**227**	scutebarbatine H	Nic	H		[78,81]
**228**	7-*O*-nicotinoylscutebarbatine H	Nic	Nic		[78,81]
**229**	scutehenanine D	Nic	Bz		[53]
Others					
**230**	scutebarbatine M	Nic	Nic	OH	[82]
**231**	scutebarbatine N	Nic	Nic	CH_2_OH	[82]
**232**	scubatine A				[55,70]
**233**	scubatine B				[70]
**234**	scubatine C				[70]
**235**	scutelinquanine B				[58]
**236**	14-deoxy-11,12-didehydroandrographolide				[60]

## Data Availability

No data was used for the research described in the article.

## Abbreviations

PI3K	Phosphatidylinositol3-kinase
AKT	Protein kinase B
mTOR	Mechanistic target of rapamycin
MAPK	Mitogen-activated protein kinase
ERK	Extracellular-regulated protein kinases
JNK	C-Jun N-terminal kinase
NFκB	Nuclear factor kappa-B
SBP	Scutellaria barbata polysaccharide
GC-MS	Gas chromatography–mass spectrometry
PLK1	Polo-like kinase 1
TP53	Tumor protein p53
BAX	BCL2-associated X
P21	Protein 21
HepG2	Human hepatocellular carcinomas
Bax	BCL2-associated X
Bcl	B-cell lymphoma-2
CNE	Human nasopharyngeal cancer cell
SBD	*Scutellaria barbata* D. Don
VEGFA	Vascular endothelial growth factor A
p-EGFR	p-Epidermal growth factor receptor
PDE7B	cAMP-specific 3′,5′-cyclic phosphodiesterase 7B
PTHrP	Parathyroid-hormone-related protein
HD-SB	*Hedyotis diffusa–Scutellaria barbata*
Smad	Drosophila mothers against decapentaplegic protein
MMP	Matrix metalloproteinase
EMT	Epithelial–mesenchymal transition
EGFR	Epidermal growth factor receptor
HUVEC	Human umbilical vein endothelial cells
ROS	Reactive oxygen species
LMO1	LIM domain only 1
PFS	Progression-free survival
OS	Overall survival
